# Genetically diverse herpesviruses in South American Atlantic coast seabirds

**DOI:** 10.1371/journal.pone.0178811

**Published:** 2017-06-02

**Authors:** Claudia Niemeyer, Cíntia Maria Favero, H. L. Shivaprasad, Marcela Uhart, Cesar Meyer Musso, María Virginia Rago, Rodolfo Pinho Silva-Filho, Paula Lima Canabarro, María Isabel Craig, Valeria Olivera, Ariel Pereda, Paulo Eduardo Brandão, José Luiz Catão-Dias

**Affiliations:** 1 LAPCOM - Laboratório de Patologia Comparada de Animais Selvagens (Wildlife Comparative Pathology Laboratory) – School of Veterinary Medicine and Animal Science, University of Sao Paulo, São Paulo, Brazil; 2 LABMAS – Laboratório de biologia Molecular Aplicada e Sorologia (Molecular Biology and Applied Serology Laboratory) – School of Veterinary Medicine and Animal Science, University of Sao Paulo, São Paulo, Brazil; 3 California Animal Health and Food Safety Laboratory System, Tulare branch, University of California Davis, Davis, California, United States of America; 4 One Health Institute, School of Veterinary Medicine, University of California Davis, Davis, California, United States of America; 5 Avidepa – Associação Vila Velhense de Proteção Ambiental, Vila Velha, Espírito Santo, Brazil; 6 Instituto de Ecologia Genética y Evolución - CONICET – Universidad de Buenos Aires, Buenos Aires, Argentina; 7 CRAM – Centro de Recuperação de Animais Marinhos – Rio Grande, Rio Grande do Sul, Brazil; 8 INTA – Instituto Nacional de Tecnología Agropecuaria – Instituto de Virología, Hurlingham, Provincia de Buenos Aires, Argentina; CEFE, FRANCE

## Abstract

Different herpesviruses have been associated with respiratory and enteric disease and mortality among seabirds and waterfowl. In 2011, a respiratory disease outbreak affected 58.3% (98/168) of the Magellanic penguins undergoing rehabilitation due to an oil spill off the southern Brazilian coast. Etiology was attributed to a novel herpesvirus identified by histopathology, immunohistochemistry, electron microscopy and molecular studies with partial DNA sequencing. Since migration, rehabilitation and translocation may facilitate the spread of pathogens between populations and trigger the onset of clinical disease in animals with latent infections, investigation of herpesvirus occurrence in asymptomatic seabirds was performed. Samples from free-ranging seabirds were collected in Argentinian Patagonia (Magellanic penguins) and the Abrolhos Archipelago in Brazil (Brown boobies, Masked boobies, Red-billed tropicbirds, White-tailed tropicbirds and South American tern). Furthermore, asymptomatic seabirds housed at the facility where the outbreak occurred were also sampled. In total, 354 samples from eight seabird species were analyzed by PCR for herpesvirus. Four different sequences of herpesviruses were identified, one in Yellow-nosed Albatross, one in Boobies and Tropicbirds and two in Magellanic penguins. Magellanic penguin herpesvirus 1 was identified during the penguin outbreak at the rehabilitation facility in Brazil, while Magellanic penguin herpesvirus 2 was recovered from free-ranging penguins at four reproduction sites in Argentina. Phylogenic analysis of the herpesviruses sequences tentatively identified suggested that the one found in Suliformes and the one associated with the outbreak are related to sequences of viruses that have previously caused seabird die-offs. These findings reinforce the necessity for seabird disease surveillance programs overall, and particularly highlight the importance of quarantine, good hygiene, stress management and pre-release health exams in seabirds undergoing rehabilitation.

## Introduction

Herpes viruses (HV) are important pathogens that have worldwide distribution and are able to infect a very wide variety of animal species, from mammals, birds, reptiles, amphibians and fishes, to oysters and clams [1]. HV cause different clinical signs and pathology depending on the virus strain and host susceptibility. Herpesvirus disease in natural hosts is often mild and followed by latent infection; however, cross-species infection may cause severe and fatal disease [2,3]. In birds, infection with HV has been identified in more than 100 different free-living species. Furthermore, the infecting ability of these viruses is currently unknown in many hosts. One might assume that natural infections present a low variability, but some HV can infect several avian species and spread within Families and even avian Orders. The *Suid herpesvirus 1*, for example, causes mild illness in pigs and other mammals yet may cause severe illness and high mortality when experimentally inoculated in chickens and pigeons [3].

Different herpesviruses have been identified as causing respiratory and enteric diseases, as well as triggering outbreaks and mortality among free-ranging marine and aquatic birds, such as *Anatid herpesvirus 1*, causing Duck plague; *Ciconiid herpesvirus 1*, in storks; *Gruid herpesvirus 1*, in cranes; *Phalacrocoracid herpesvirus 1*, in cormorants; *Fregata magnificens herpesvirus 1*, in frigatebirds; and *Gaviid herpesvirus 1* in loons [1, 3, 4, 5]. Due to their frequency and relevance, Kaleta *et al*. (2007), suggest that HV investigations should be incorporated into wild bird disease surveillance programs.

A herpesvirus-like infection affecting African penguins was first described at two sites, the Baltimore Zoo [6] and at a rehabilitation facility in South-Africa [7]. In both cases the authors suggested a herpesvirus infection due to clinical and histopathological alterations, but virus isolation and DNA sequencing was not achieved. In fact, herpesvirus infection has thus far not been confirmed in Magellanic penguins (*Spheniscus magellanicus*), nor in free-ranging penguins of any species.

In this article, we report an outbreak in rescued Magellanic penguins due to a possibly novel herpesvirus causing severe haemorrhagic respiratory disease. Moreover, we report the detection and characterization of sequences of what could be four novel herpesvirus in five different seabirds species sampled in coastal areas off the South Atlantic. As birds from different populations can interact naturally during migration and because of humans during rehabilitation, potentially favoring the spread of pathogens between populations and species, these two findings complement each other. Herpesvirus findings in asymptomatic seabirds might help understand the origin and contributing factors leading to outbreaks in captivity.

## Materials and methods

### Respiratory disease outbreak in Magellanic penguins undergoing rehabilitation

In the Brazilian winter season of 2011, an outbreak of acute respiratory disease occurred in CRAM (Centro de Recuperação de Animais Marinhos), a rehabilitation centre located in Southern Brazil (32°02’06”S, 52°05’55” W). A total of 168 oiled Magellanic penguins, 17 adults and 151 juveniles (first year plumage), were rescued after stranding along the Rio Grande do Sul State beaches. Between mid-June to mid-July, 98 (58.3%) penguins developed acute respiratory signs and 85 of them died. Thirteen penguins were humanely euthanized with 2mL IV of T61^™^ (MSD Saúde Animal – Sao Paulo/Brazil) due to severe dyspneic condition. A total of 89 samples were PCR analyzed, including 52 macerated trachea tissue samples from necropsied animals and 37 tracheal swabs from live ill animals.

#### Pathology

Sixty-seven penguins were necropsied. Tissue samples were fixed in 10% formalin solution for 48 hours and sent to the Wildlife Comparative Pathology Laboratory, Veterinary Medicine School and Animal Science, University of São Paulo (LAPCOM-FMVZ, USP) for histopathological analysis. The samples were cut, embedded in paraffin, sectioned at a thickness of 4μm, stained with hematoxylin and eosin (H.E.) and analyzed under optical microscope. Selected slides containing air sacs and lung cuts were stained with PAS (Periodic acid-Schiff) for differential fungal infection diagnosis.

#### Electron microscopy

Portions of 1 mm^3^ of tracheal mucosa from two penguins previously fixed in a 10% formalin solution and embedded in paraffin were deparaffinised and fixed in 2.5% glutaraldehyde solution, post fixed with osmium tetroxide, dehydrated and finally embedded in Epon. Ultrathin sections (70 μm) were stained with uranyl acetate and lead citrate and examined by transmission electron microscopy according to the protocol described by Doane and Anderson, (1987) [8].

#### Immunohistochemistry

The protocol used for immunohistochemistry was described by Guy et al. (1992). Portions of tracheal tissue fixed and paraffined samples from the same two penguins pictured by electron microscopy were sliced with a thickness of 4 microns and placed on slides. Slides were deparaffinised using xylene and rehydrated by washing with alcohol in various degrees of dilution. After rehydration, hydrated hydrogen peroxide was used to inhibit peroxidase. The antigen retrieval was performed by heating the slides in citrate buffer (Decloaker Diva, Biocare Medical, Concord, CA) for 10 minutes in an autoclave (Decloaker). The return of pressure and cooling of the slides was performed with the gradual addition of deionized water. After two washes with TBS-Tween solution (Tris buffer saline with Tween 20), unspecific binding regions were blocked with the use of commercial casein-based solution (Background Punisher, Biocare Medical, Concord, CA). Murine monoclonal antibody directed against *Gallid herpesvirus 1* (A1) (kindly provided by Dr. J. Guy) was added in a dilution of 1: 4000 for 30 minutes at room temperature. After washing with TBS-Tween solution, the slides were incubated with polymer anti-mouse HRP-labeled (HRP Envision + System Polymer Labeled Anti-Mouse, Dako, Carpinteria CA) for 30 minutes at room temperature. Substrate chromogen 3-amino-9-ethylcarbazole (AEC Substrate Chromogen, Ready-to-Use, Dako, Carpinteria CA) was added to the slides for 10 minutes followed by washing with TBS-Tween solution. The slides were then stained with Mayer Hematoxylin and prepared with aqueous assembly and the permanent coverslip was placed after drying the solution [9].

### Asymptomatic seabird survey

#### Herpes virus survey in free-ranging seabirds at reproductive colonies

A cross-sectional survey of free-ranging seabirds in the Abrolhos Archipelago (17°25´ to 18°09´S and 38°33´ to 39°05´W), located in the northeastern Brazilian Coast, was undertaken in March 2013. We sampled breeding boobies (*Sula leucogaster* and *Sula dactylatra*), tropicbirds (*Phaeton aethereus* and *Phaeton lepturus*) and a tern (*Sterna hirundinacea*). In addition, we sampled Magellanic penguins at four breeding colonies in Argentina (Península Valdés– 41°43´S/62°33´W; Punta Tombo—44°04´S/65°11´W; Cabo Dos Bahías—44°55´S/65°33´W; Bahía Bustamante—45°08´S/66°32´W) in January 2014.

#### Herpes virus survey in non-penguin seabirds undergoing rehabilitation at CRAM

Samples were collected from apparently healthy rescued seabirds as they were admitted to the CRAM rehabilitation centre (Centro de Recuperação de Animais Marinhos, Rio Grande, RS State; 32°1'60''S/52°5'55''W). Two Southern Giant Petrels (*Macronectes giganteus)* were sampled at the time of the penguin outbreak. In March 2013 (20 months later), we sampled twelve Atlantic yellow-nosed albatrosses (*Thalassarche chlororhynchos*) recovered from a mass stranding.

Overall, samples were collected from 265 asymptomatic individual seabirds of eight species (Table 1). Tracheal cotton or polyester swabs were collected from live birds that were manually restrained, sampled and released. The swabs were dry stored and frozen in liquid nitrogen within 4–8 hours of collection, and later kept in an ultralow freezer at -80°C until analyzed.

**Table 1:** Number, species, location and percentage positive for asymptomatic seabirds screened for herpesvirus DNA.

	Taxonomic family	Common name	Latin name	Location	Age	No. of positives /birds screened (%)	Potentially detected herpesvirus
**Free-ranging**	Sulidae	White booby	*Sula dactylatra*	Abrolhos—BR	Adult	2/23 (8.6%)	SuHV
	Sulidae	Brown booby	*Sula leucogaster*	Abrolhos—BR	Adult	1/20 (5%)	SuHV
	Phaethontidae	Red billed tropicbird	*Phaeton aethereus*	Abrolhos—BR	Adult	2/29 (6.8%)	SuHV
	Phaethontidae	White-tailed tropicbird	*Phaeton lepturus*	Abrolhos—BR	Adult	0/4	
	Sternidae	South American tern	*Sterna hirundinacea*	Abrolhos—BR	Adult	0/1	
	Spheniscidae	Magellanic penguin	*Spheniscus magellanicus*	Península Valdés—AR	Adult	0/36	MagHV-2
	Spheniscidae	Magellanic penguin	*Spheniscus magellanicus*	Península Valdés—AR	Nestling	1/5	MagHV-2
	Spheniscidae	Magellanic penguin	*Spheniscus magellanicus*	Punta Tombo—AR	Adult	0/35	MagHV-2
	Spheniscidae	Magellanic penguin	*Spheniscus magellanicus*	Punta Tombo—AR	Nestling	2/10	MagHV-2
	Spheniscidae	Magellanic penguin	*Spheniscus magellanicus*	Cabo Dos Bahías—AR	Adult	2/35	MagHV-2
	Spheniscidae	Magellanic penguin	*Spheniscus magellanicus*	Cabo Dos Bahías—AR	Nestling	1/10	MagHV-2
	Spheniscidae	Magellanic penguin	*Spheniscus magellanicus*	Bahía Bustamante—AR	Adult	2/36	MagHV-2
	Spheniscidae	Magellanic penguin	*Spheniscus magellanicus*	Bahía Bustamante—AR	Nestling	1/6	MagHV-2
**Rehabilitation**	Diomedeidae	Atlantic yellow-nosed albatross	*Thalassarche chlororhynchos*	CRAM—BR	Adult	1/12 (8.3%)	ThaHV
	Procellariidae	Southern giant petrel	*Macronectes giganteus*	CRAM—BR	Juvenile	0/2	
	Spheniscidae	Magellanic penguin	*Spheniscus magellanicus*	CRAM—BR	Juvenile	34/89 (38%)	MagHV-1

#### Polymerase chain reaction (PCR) and phylogenetic analysis

Samples from penguins in Argentina were analyzed at the Virology Laboratory of the National Agricultural Technology Institute (INTA). Samples were pooled in groups of 4 to 5 penguins of same age category and location. Viral DNA was extracted from pooled tracheal swabs by using a QIAamp^®^ DNA Mini kit (QIAGEN^®^, Hilden, Germany) following the manufacturer´s instructions. Samples collected in Brazil were evaluated at the Molecular Biology and Applied Serology Laboratory (LABMAS) of the University of Sao Paulo. Viral DNA was extracted using a phenol/chloroform protocol. PCR was used to detect diverse herpesvirus as previously described by VanDevanter *et al*. (1996). The degenerated primers DFA (5´-GAYTTYGCNAGYYTNTAYCC-3´), ILK (5´-TCCTGGACAAGCAGCARNYSGCNMTNAA-3´) and KG1 (5´-GTCTTGCTCACCAGNTCNACNCCYTT-3´) targeted the DNA polymerase protein, UL30 gene region, which is quite conserved among all known herpesvirus types [10]. This procedure was followed by a nested-PCR with the primers TGV (5´-TGTAACTCGGTGTAYGGNTTYACNGGNGT-3´) and IYG (5´-CACAGAGTCCGTRTCNCCRTADAT-3´) under the same reaction conditions as the initial PCR, which had an expected product size of 215 to 315bp. In an attempt to acquire a longer 460pb sequence, PCR products using KG1 and TGV primers were purified by using a commercial purification kit (ExoSAP-IT^®^; USB^®^, Piscataway—NJ, USA) according to the manufacturer’s instructions and were sequenced by using BigDye^®^ Terminator v3.1 commercial kit (Applied Biosystems^®^, Austin—TX, USA) as recommended by the manufacturer (ABI Prism^®^ 310 Genetic; Applied Biosystems^®^, Foster City—CA, USA).

Nucleotide sequences derived from this study have been deposited in the Genbank sequence database. Sequences were aligned with previously published herpesvirus sequences obtained from Genbank by using the Clustal program within the BioEdit Sequence Alignment Editor and MEGA 6.0 software package [11]. Phylogenetic analyses were conducted in MEGA 6.0 and the evolutionary history was inferred by using the Maximum Likelihood method based on the Le Gascuel 2008 model. Initial tree(s) for the heuristic search were obtained automatically by applying Neighbor-Join and BioNJ algorithms to a matrix of pairwise distances estimated using a JTT model, and then selecting the topology with superior log likelihood value. A discrete Gamma distribution was used to model evolutionary rate differences among sites (5 categories (+G, parameter = 0.7729)). The rate variation model allowed for some sites to be evolutionarily invariable ([+I], 12.9113% sites). The analysis involved 26 amino acid sequences. All positions containing gaps and missing data were eliminated. There were a total of 47 positions in the final dataset [12,13].

All procedures used for sampling free-ranging and in rehabilitation birds were approved by the Ethics Committee on the Use of Animals of the School of Veterinary Medicine and Animal Science of University of São Paulo, and were authorized by the Environmental Ministry under permit number 36250–5. Argentinian permits were issued by Ministerio de Agricultura, Ganaderia, Bosques y Pesca (resolutions 5442/10 and 99/11).

## Results

### Respiratory disease outbreak in Magellanic penguins undergoing rehabilitation

Of 168 oil-spill rescued penguins, 98 (58.3%) became ill. Clinical signs including anorexia, gasping, coughing, expectoration of bloody mucus and marked dyspnoea were observed in all of the affected birds. Fourteen penguins died within 24 hours of arrival to the rehabilitation center, but most died within a median of two weeks after onset of clinical signs. The deaths occurred between June 22nd and July 14^th^, 2011. All the birds that presented clinical signs died or were euthanized due to severe respiratory distress. The prognosis was poor even with antibiotic treatment (125mg/kg amoxicillin/clavulanic acid BID). In an attempt to control the outbreak, all birds presenting clinical sign were placed in isolation and strict measures of hygiene and disinfection of premises and fomites were implemented. Only veterinarians and selected staff accessed and cared for the sick penguins. In addition, penguins with no clinical signs were housed in more open and ventilated enclosures in a distant facility.

**Fig 1:**
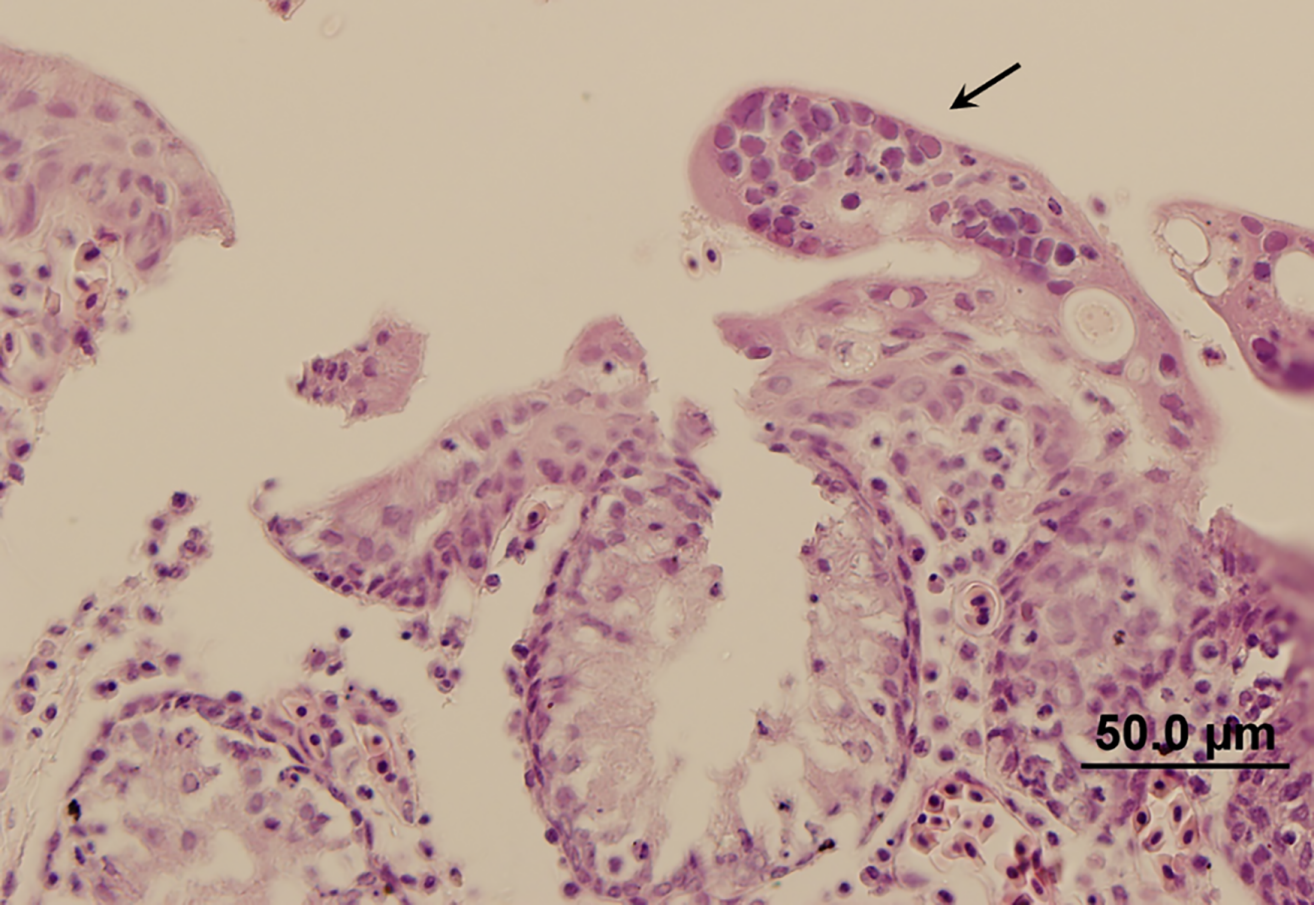
H&E slide of trachea epithelium with multiple foci of necrosis and squamous metaplasia. Syncytial giant cells in the tracheal epithelium of Magellanic penguins contained amphophilic intranuclear inclusions (black arrow) characteristic of Herpesvirus infection. H&E.

All affected birds were considered to be in fair to poor nutritional condition based on lack of adipose tissue and low body weights (average 2.5kg; range 1.7 to 4.3 kg). Necropsy revealed that all penguins had various degrees of respiratory lesions; 41% (28/67) had necro-haemorrhagic tracheitis, 83% (56/67) fibrinous airsacculitis, and 86% (58/67) had oedema and congestion of the lungs. Furthermore, 20.9% (14/67) presented with splenomegaly and 14.9% (10/67) with hepatomegaly in addition to respiratory lesions. Histopathology revealed severe hyperplasia and fibrionecrohemorrhagic tracheitis associated with syncytial cell formations, many of which contained eosinophilic intranuclear inclusions (Fig 1). All penguins had severe pulmonary congestion and multifocal to coalescing haemorrhagic lesions. Acute, diffuse heterophilic pneumonia was noticed in 86% (58/67) of the analyzed tissues and 17.9% (12/67) showed pyogranulomatous pneumonia associated with fungi. Membranous airsacculitis was verified in 83.5% (56/67) of the analyzed cases and intranuclear inclusion bodies were seen in the thickened air sacs.

**Fig 2:**
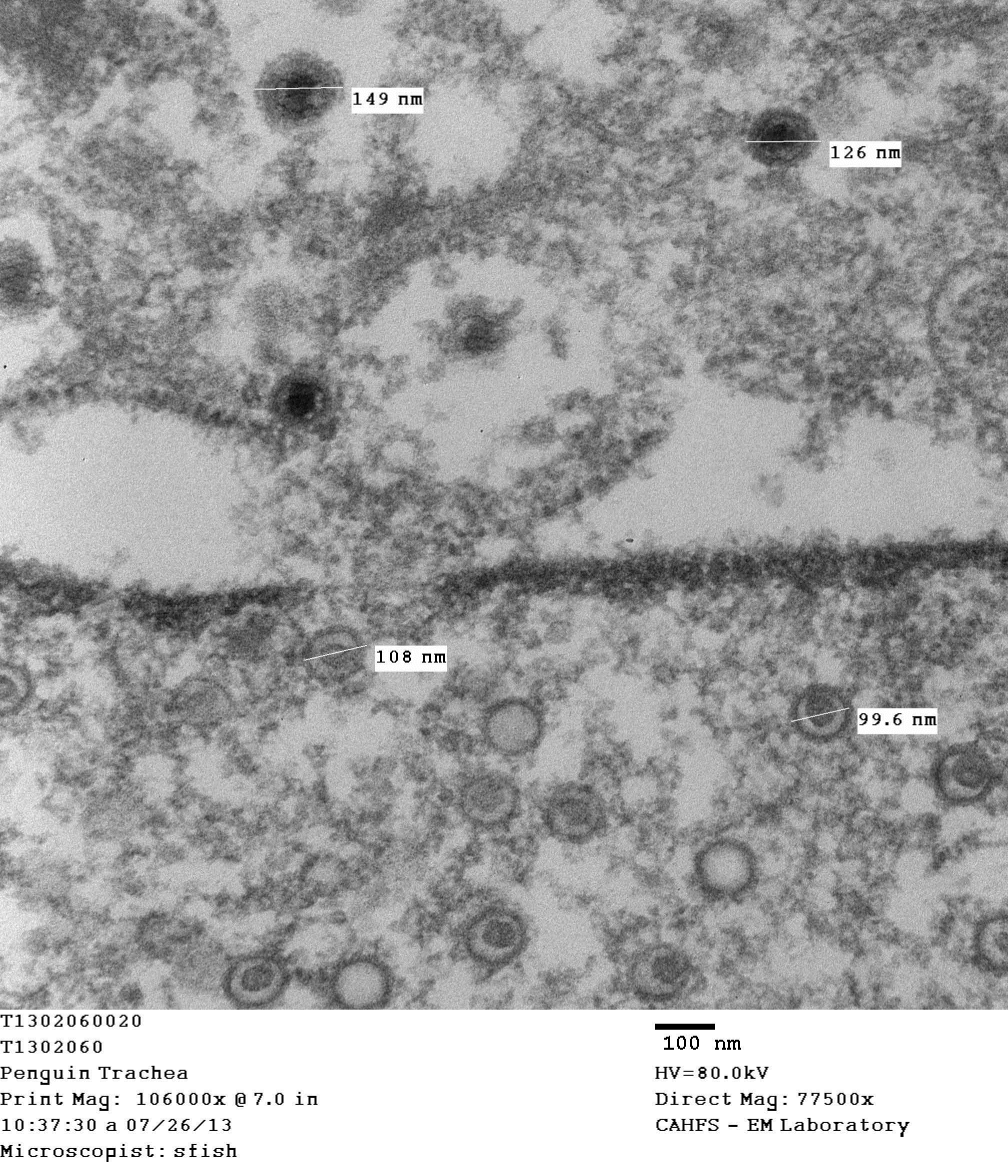
Electron microscopy of Magellanic penguin tracheal epithelial cell. The epithelial cell nuclei with intranuclear viral particles and enveloped virions in the extracellular space from a Magellanic penguin (*Spheniscus magellanicus*).

Electron microscopy of paraffin-embedded trachea samples showed that the nuclear inclusions observed by light microscopy consisted of dense aggregates of viral particles. Viral enveloped capsid particles as well as empty envelopes were mainly seen in the nucleus and their size ranged from 99.6 to 101nm and in the cytoplasm ranged from 126 to 149 nm including the envelop (Fig 2).

**Fig 3:**
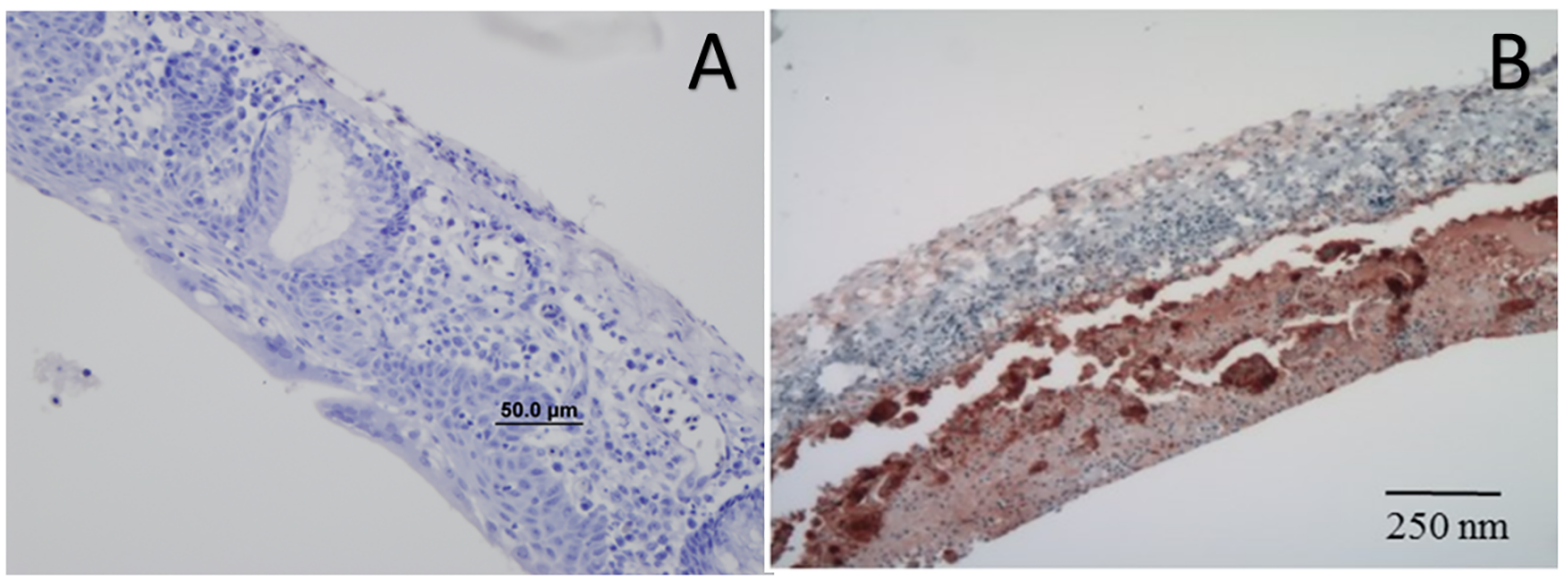
Immunohistochemical assay of Magellanic penguin (*Spheniscus magellanicus*) trachea tissue. (A) Negative immunohistochemical assay of Magellanic Penguin (*Spheniscus magellanicus*) trachea tissue using specific anti-Gallid herpesvirus 1 murine monoclonal antibody and (B) positive control of trachea from chickens (*Gallus gallus*) naturally infected with GaHV-1, showing intense coloration where reaction occured. Hematoxylin Mayer.

Immunohistochemistry on paraffin embedded tracheas for chicken Infectious Laryngotracheitis (ILT) virus was negative, suggesting that even though the histopathological lesions resembled those of ILT, and the electron microscopy confirmed the presence of a herpesvirus, it was not *Gallid herpesvirus 1* (GaHV1) (Fig 3).

Eighteen (18/52) macerated trachea tissues and 16 (16/37) tracheal swabs tested by PCR were positive and their products were sequenced. An expected fragment size of 460bp regarding the partial sequence of the herpesvirus DNA polymerase gene was detected 15 times in different positive Magellanic penguin samples.

### Asymptomatic seabird herpes virus survey

Herpesvirus DNA was detected in 15 (5.6%) of the 265 tracheal swabs analyzed. All animals sampled in the colonies were apparently healthy. Herpesvirus sequence findings by species and site are shown in Table 1. Fragments of 460 bp of the partial sequence of the UL30 gene encoding the DNA polymerase of herpesviruses were detected in five different seabird species. DNA from partial polymerase herpesvirus gene sequences were obtained in samples from masked booby, brown booby, red-billed tropicbird, yellow nosed-albatross in Brazil, and Magellanic penguin in Argentina. A total of nine sequences were obtained from all seabirds and sequences belonging to the same genotype presented no divergence.

**Fig 4:**
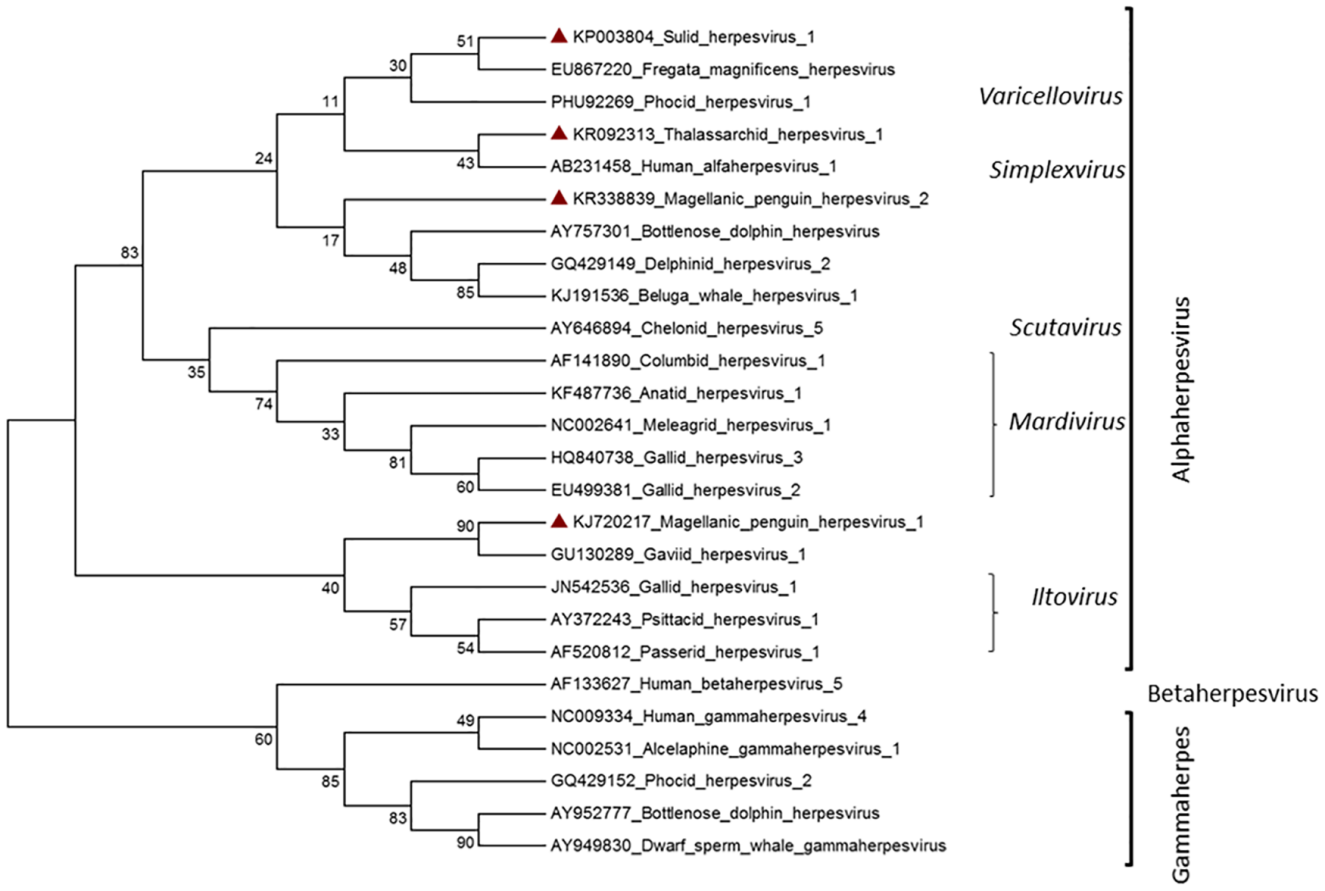
Molecular phylogenetic analysis by Maximum Likelihood method. The evolutionary history was inferred by using the Maximum Likelihood method based on the Le_Gascuel_2008 model [13]. The tree with the highest log likelihood (-1242.2246) is shown. The tree is drawn to scale, with branch lengths measured in the number of substitutions per site. The analysis involved 26 amino acid sequences. Evolutionary analyses were conducted in MEGA6 [11].

Phylogenetic analysis was done by comparing the herpesvirus sequences obtained from the penguin outbreak and the asymptomatic seabird survey, with 26 other sequences available in Genbank. The choice of sequences was based on standard viral sequences that characterize subfamilies according to the ICTV [12], and herpesvirus sequences that were not yet classified, but which have been identified in seabirds and marine mammals (Fig 4). All herpesviruses sequences identified in this study were classified as belonging to the Alphaherpesvirinae subfamily. As ours were the first deposited sequences in Genbank of these seabird genuses, we suggest that the genotype identified in boobies could be named as Sulid herpesvirus (SuHV) genbank accession number KP003804; Thalassarchid herpesvirus (ThaHV) genbank accession number KR092313, for the virus found in yellow-nosed albatross, and Magellanic penguin herpesvirus 1 (MagHV-1) genbank accession number KJ720217 and Magellanic penguin herpesvirus 2 (MagHV-2) genbank accession number KR338839 for genotypes identified in Magellanic penguins in Brazil and Argentina, respectively.

## Discussion

Viral findings in free-living seabirds can be associated with the occurrence of mortality in breeding colonies or rehabilitation centers. In the present study, we identified herpesvirus sequences both in sick birds that were undergoing rehabilitation, as well as in apparently healthy birds which were active at their reproductive sites, in which the virus was probably in a latent state. We report sequences that could correspond to four novel seabird herpesviruses identified for the first time in hosts such as Magellanic penguin, masked booby, brown booby, red-billed tropicbird and albatross. Penguin samples harbored two different herpesvirus genotypes, though no bird was co-infected with more than one genotype at the time.

### Respiratory disease outbreak in Magellanic penguins undergoing rehabilitation

Gross and microscopic findings, electron microscopy and molecular analysis were consistent with herpesvirus infection in birds. The pattern of lesions found in penguins, including the presence of intranuclear inclusion corpuscles and clusters of syncytial cells with amphophilic core, is consistent with the pattern of disease described for the Iltovirus genus, such as *Gallid herpesvirus 1* (GaHV -1) or laryngotracheitis virus of chickens, and *Passerid herpesvirus 1* (PaHV-1) or tracheitis-causing virus in Gouldian Finch (*Erythrura gouldiae*), which was also considered in the phylogenetic analysis (Fig 4) [2, 14]. Although a herpesvirus-like infection had been described in captive African penguins in the Baltimore Zoo, United States [6], and in South Africa [7], the occurrence of herpesvirus infection in penguin species is likely uncommon. The CRAM rehabilitation centre in Brazil has operated for the past 30 years without any similar mortality outbreak as the one reported here.

In our study, the sequence corresponding to Magellanic penguin herpesvirus I, MagHV-1, (Genbank KJ720217) was identified at CRAM rehabilitation centre in July 2011, and caused the death of more than 58.3% of the rescued penguins that year. The sequence corresponding to Magellanic penguin herpesvirus 1 grouped with Gaviid herpesvirus 1 (GavHV-1), a virus which was associated with respiratory disease in large loons (*Gavia immer*) undergoing rehabilitation off the coast of Florida in the United States [5]. Both in the Florida rehabilitation centre [5] and in southern Brazil (this investigation), secondary bacterial and fungal infections were observed in affected birds, which worsened their general state and contributed to mortality.

Herpesviruses commonly have a high prevalence in natural hosts and establish latent infections for life in the trigeminal nerve ganglia [3, 15]. Viral reactivation usually occurs in immunosuppressed individuals and may be associated with high levels of stress such as maintenance in captivity or intoxication by chemical pollutants [5, 16]. Although the origin of MagHV-1 sequences described in this study is unknown, the acute mortality pattern observed suggests that most of the animals had no previous contact with this viral genotype. Our hypothesis is that a few birds presented the virus in a latent state and developed clinical disease due to the immunosuppressive effects of both oil contamination and rehabilitation distress. Infection could have then quickly disseminated among penguins due to high-density aggregation in captivity. Our findings suggest that further investigations of herpesviruses in healthy, free ranging Magellanic penguins, and systematic monitoring at seabird rehabilitation centres should be conducted to improve our understanding of herpesvirus occurrence and prevalence.

### Asymptomatic seabird herpes virus survey

#### Herpes virus survey in free-ranging seabirds at reproductive colonies

In January 2014, two and a half years after the respiratory disease outbreak, we identified sequences corresponding to Magellanic penguin herpesvirus 2, MagHV-2, (Genbank KR338839) in both nestling and adult Magellanic penguins, at breeding colonies in Argentinian Patagonia. Sequences of Magellanic penguin herpes 2 genotype found in breeding penguins at Argentinian colonies was distinct (50% similarity) from sequences of Magellanic penguin herpes 1 identified during the respiratory outbreak at CRAM. Even though no bird was co-infected with more than one genotype at the time, we cannot rule out the possibility that there might exist more than one herpesvirus genotype adapted to penguins, as has been demonstrated in dolphins by Benson et al. (2006) [17].

The sequences corresponding to Sulid herpesvirus, SuHV (Genbank KP003804) was identified in five apparently healthy birds, including two masked boobies, one brown booby and two red-billed tropicbirds which had chicks in their nests at the Santa Barbara Island (Abrolhos Archipelago—Brazil). These sequences showed 65% similarity to *Frigatebird herpesvirus 1* (FmagHV-1) identified in frigatebirds in French Guinea [4], and 76% similarity to *Vulture herpesvirus* (VHV), identified in vultures in Asia [18]. These two previously reported viruses were recovered from carcasses, and the frigatebird genotype caused skin and bone disease and a big die-off in free-ranging birds [4]. Phylogenetic analysis showed that SuHV grouped together with the FmagHV-1 and VHV-1. Thus, it is expected that if birds were to express any type of clinical signs, they would be related to the skin pattern. The Herpesvirus previously described in frigatebirds was identified in Grand Connétable Islands (4°49'30''N, 51°56'00''W), a group of rocky islands located near the equator in the North Atlantic, the only nesting site for the species between Tobago (11° 00´ N, 61° 00 W) and Fernando de Noronha, Brazil (3°51'13″ N, 32°25'25″ W). Although frigatebirds are not migratory nor fish in high seas, they interact extensively with other seabirds forcing them to regurgitate their food and stealing it. Thus, frigatebirds usually eat saliva and body fluids of other birds and could easily become infected by this route [4,19]. At the Abrolhos Archipelago where the samples in our study were collected, there is also a nesting colony of frigatebirds in Redonda Island. Thus far, that population has not been sampled for viruses and it is unclear whether it might be infected by herpes. Even though no herpesvirus related clinical signs have been observed since 2011 when we started monitoring the health of the birds in the Archipelago, a sampling expedition in planned for the next breeding season for follow up.

#### Herpes virus survey in seabirds undergoing rehabilitation at CRAM

The Thalassarchid herpesvirus, ThaHV, (Genbank KR092313) was identified from one of 12 yellow-nosed albatrosses in rehabilitation. The bird was rescued from a beach in Rio Grande do Sul State during an unusual mass stranding of 125 Albatrosses in March 2013 reported by Faria *et al* (2014) [20,21]. The twelve rescued birds were found dehydrated and taken to CRAM. After receiving basic care, the birds met CRAM criteria for release within three days and were set free. Unfortunately we did not have access to 113 remaining Albatross carcasses on the beach, and it is therefore not possible to determine whether the herpesvirus played a role in the mass stranding. Further herpesvirus investigations should be conducted in Albatrosses.

## Conclusion

The the phylogeny based on DNAPOL amino acid sequences showed that outbreak-associated MagHV-1 sequences grouped with *Gaviid herpesvirus 1* (GavHV-1) and within the genus *Iltovirus*. This is consistent with clinical signs and lesions associated with this virus resembling those of chicken infectious laryngotracheitis caused by *Gallid herpesvirus 1*. Based on the same analyses, the Thalassarchid herpesvirus (ThaHV) and the Magellanic penguin herpesvirus 2 (MagHV-2) sequences were grouped between human herpesvirus *Simplex* genus, and some marine mammals herpesviruses that are still classified as unassigned genus. The herpes identified in Suliformes (SuHV-1) was tentatively grouped with fregata herpesvirus and phocine herpesvirus within the *Varicellovirus* genus. Percent bootstrap support for these groups was 30 or less however.

Herpesviruses usually have a high prevalence in their natural host and establish latent infections throughout life [3]. Prevalence in this study ranged from 5 to 50%, but because we pooled samples collected in Argentina, and only obtained a small number of samples from some species, we cannot establish infection rates. The authors also acknowledge the limitations on the detection of herpesviruses by the sampling methods used and by the potential for latent infection (and consequent lack of detection) in the species studied. Representative studies of each population should be conducted to address this issue, perhaps by initially screening stored serum samples from these same individuals for specific antibodies. However, the fact that we found about 5% of sampled birds to be positive in an initial random screening along the south Atlantic coast suggests that its occurrence in seabird populations is possibly high. Further studies based on longer sequences of the viruses found in this study and their infectivity for other bird species are ongoing, and will allow for a better understanding of the origin and potential risk of these virus strains.

Samples in our study were collected from wild bird populations comprising eight species of seabirds from diverse habitats, and herpesvirus DNA was detected in each surveyed area. HVs have been previously detected in seabirds worldwide, but thus far have never been reported in South Atlantic seabird populations. All breeding seabirds from which herpesviruses were detected in this study appeared to be healthy. Herpesvirus are known to stay in latent state in adapted host species. It is also known that the occurrence of outbreaks and mortality in wild bird populations may be triggered by immunosuppression, which can be caused by acute or chronic habitat pollution or degradation and/or be associated with other infectious agents [3,4,18]. Having identified the presence of latent herpesviruses in marine species inhabiting the South American Atlantic coast, we can expect that disturbances, whether environmental or physiological and leading to immunosuppression, may predispose them to disease outbreaks and mortality. Our results highlight the need for sustained monitoring of diseases in seabird populations, especially in light of planed intensification of oil exploration in the South West Atlantic continental shelfs.
